# Oxidative Stress in Cancer Cell Metabolism

**DOI:** 10.3390/antiox10050642

**Published:** 2021-04-22

**Authors:** Saniya Arfin, Niraj Kumar Jha, Saurabh Kumar Jha, Kavindra Kumar Kesari, Janne Ruokolainen, Shubhadeep Roychoudhury, Brijesh Rathi, Dhruv Kumar

**Affiliations:** 1 Amity Institute of Molecular Medicine and Stem Cell Research (AIMMSCR), Amity University Uttar Pradesh, Sec 125, Noida 201303, India; saniya.arfin@s.amity.edu; 2 Department of Biotechnology, School of Engineering & Technology (S.E.T.), Sharda University, Greater Noida 201310, India; niraj.jha@sharda.ac.in (N.K.J.); saurabh.jha@sharda.ac.in (S.K.J.); 3 Department of Applied Physics, School of Science, Aalto University, 00076 Espoo, Finland; kavindra.kesari@aalto.fi (K.K.K.); janne.ruokolainen@aalto.fi (J.R.); 4 Department of Life Science and Bioinformatics, Assam University, Silchar 788011, India; shubhadeep1@gmail.com; 5 Laboratory for Translational Chemistry and Drug Discovery, Department of Chemistry, Hansraj College, University of Delhi, New Delhi 110021, India; brijeshrathi@hrc.du.ac.in

**Keywords:** mitochondrial ROS, oxidative stress, cancer metabolism, warburg effect, tumor progression, apoptosis, autophagy, NFκB pathway, tumor adaptation, drug resistance, angiogenesis, metastasis, tumor targeting

## Abstract

Reactive oxygen species (ROS) are important in regulating normal cellular processes whereas deregulated ROS leads to the development of a diseased state in humans including cancers. Several studies have been found to be marked with increased ROS production which activates pro-tumorigenic signaling, enhances cell survival and proliferation and drives DNA damage and genetic instability. However, higher ROS levels have been found to promote anti-tumorigenic signaling by initiating oxidative stress-induced tumor cell death. Tumor cells develop a mechanism where they adjust to the high ROS by expressing elevated levels of antioxidant proteins to detoxify them while maintaining pro-tumorigenic signaling and resistance to apoptosis. Therefore, ROS manipulation can be a potential target for cancer therapies as cancer cells present an altered redox balance in comparison to their normal counterparts. In this review, we aim to provide an overview of the generation and sources of ROS within tumor cells, ROS-associated signaling pathways, their regulation by antioxidant defense systems, as well as the effect of elevated ROS production in tumor progression. It will provide an insight into how pro- and anti-tumorigenic ROS signaling pathways could be manipulated during the treatment of cancer.

## 1. Introduction

Reactive oxygen species (ROS), the partially reduced metabolites of oxygen that possess strong oxidizing capabilities, are deleterious to cells at high concentrations but at low concentrations, they serve complex signaling functions. Reactive oxygen species formed as byproducts of normal cell metabolism are needed for maintaining homeostasis and cellular signaling. Apart from cellular metabolism they are generated by specific plasma membrane oxidases in response to growth factors and cytokines and serve as secondary messengers in specific signaling pathways and play a role in regulating gene expression [1]. Cells have a defense system to maintain ROS at physiologically normal levels, i.e., enzymes called antioxidants, responsible for transforming free radicals into stable, less damaging molecules, the impairment of which may lead to a state of oxidative stress [2]. These oxygen scavenging pathways include conversion of O_2_^−^ to H_2_O_2_ by superoxide dismutase (SOD), the action of catalase on H_2_O_2_ to produce H_2_O and O_2_, decomposition of H_2_O_2_ and LOOH by Glutathione peroxidase, and the reduction of H_2_O_2_ by Thioredoxin reduction cycle to produce H_2_O and also the exogenous detoxification of glutathione transferase [3]. Cancer cells are highly metabolically active and hypoxic cells, and due to massive growth and insufficient vascular irrigation tend to produce increased ROS, which damages DNA by diffusing through the mitochondrial membrane while also acting as signal-transducing messengers in many redox-sensitive molecular pathways involved in cell survival, therapeutic resistance, and progression [3]. Oxidative stress plays a major role in cancer hallmarks like angiogenesis, invasiveness, stemness, and metastatic ability, and hence, reducing oxidative stress with powerful antioxidants has been used as an important strategy for cancer prevention. Additionally, cancer cells develop mechanisms of keeping the increased oxidative stress in check. Therefore, some cancer therapeutic strategies also work by disrupting this check and making the cancer cells susceptible to apoptosis.

## 2. Source of Reactive Oxygen Species in Cancer Cells

### 2.1. Mitochondrial ROS

Mitochondria is one of the most prominent sources of reactive oxygen species within a cell which contribute to oxidative stress [4]. The electron transport chain located on the inner mitochondrial membrane generates the majority of mitochondrial ROS during the process of oxidative phosphorylation (OXPHOS). Leakage of electrons at complex I and complex III from ETC leads to a partial reduction of oxygen to form superoxide which undergoes spontaneous dismutation to hydrogen peroxide, both of which are collectively considered as mitochondrial ROS [5]. Endogenous modulators such as NO and Ca^2+^ have been observed to regulate the production of mtROS by regulating the metabolic states of mitochondria. The mitochondrial Ca^2+^ levels increase the rate of electron flow in the ETC and thus decrease mtROS generation [6]. However mitochondrial Ca^2+^ overload increases mtROS production [7]. STAT3, a transcription factor that regulates gene expression in response to cytokines interleukin (IL)-6 and IL-10, also modulates the activity of the ETC [8,9]. Hence a decrease in expression of STAT3 may be correlated to increasing mtROS at complex I [8]. TNF-α that causes the shedding of TNF-α receptor-1 reducing the severity of microvascular inflammation, has been found to induce a calcium-dependent increase in mt ROS [10]. Studies have shown that many ROS-producing enzymes, like NADPH oxidase, xanthine oxidase, and uncoupled eNOS, can stimulate mtROS production in a process called “ROS-induced ROS” [11,12,13]. Another transcription factor hypoxia-inducible factor 1α (HIF-1α) also plays a prominent role in bringing about a reduction in ROS by a number of mechanisms including induction of pyruvate dehydrogenase kinase 1 (PKD1), which shunts pyruvate away from the mitochondria; triggering mitochondrial selective autophagy; and induction of microRNA-210 blocking OXPHOS [14]. Low levels of mtROS regulate the stability of HIF-1α leading to hypoxia adaptation while moderate levels of mtROS have been found to regulate the production of proinflammatory cytokines by directly activating the inflammasome and mitogen-activated protein kinase (MAPK). However, high levels of mtROS are capable of inducing apoptosis by oxidation of the mitochondrial pores and autophagy by the oxidation of autophagy-specific gene 4 (ATG4) [5]. Depending on the tumor cell microenvironment, the c-Myc gene controls apoptosis by inducing aerobic glycolysis and/or OXPHOS which is required for the activation of certain tumor suppressor proteins, such as Bax and Bak [15,16,17].

Mitochondria also play an important role in the loss of caveolin 1 (cav-1) in the tumor-associated fibroblast compartment, which is related to the early tumor recurrence, metastasis, tamoxifen-resistance, and aggravated increase in tumor growth [2]. Cav-1 loss induces autophagy and mitophagy, [18] driving the “Reverse Warburg Effect” by a feed-forward mechanism. This onset of inflammation, autophagy, mitophagy, and aerobic glycolysis in the tumor microenvironment is triggered by activation of the transcription factors NFκB and HIF-1α [19,20]. Mitochondria-generated ROS plays an important role in cell proliferation and quiescence. The pro- or anti-tumorigenic signaling is controlled by a mitochondrial ROS switch of the antioxidant SOD2/MnSOD [21]. Cell proliferation is favored by decreased SOD2/MnSOD activity resulting in increased O_2_^−^ production whereas proliferating cells transit into quiescence when SOD/MnSOD activity increases resulting in increased H_2_O_2_ activity [22]. Inactivation of mitochondrial antioxidant responses like the Thioredoxin reductase (TrxR); which causes reduction of oxidized Trx to produce reduced Trx that reacts with ROS, contributes to increased oxidative stress in cancer cells.

Studies have shown that the cellular redox status is impacted by the recruitment of mitochondria by the expression of hTERT. This observation is supported by the presence of hTERT in the mitochondria and since mitochondrial-dependent apoptosis in target cells can be carried about by introducing hTERT inhibitors [23].

### 2.2. Role of Warburg Effect in ROS

The increased metabolic requirements of the cancer cells are met by upregulation of glucose transport and metabolism irrespective of oxygen supply [24]. There is also some evidence that cancer cells decrease mitochondrial respiration in the presence of oxygen, which suppresses apoptosis [25]. Under hypoxic conditions, the accelerated metabolism produces ROS in cancer cells that is countered by the increased NADPH which is met by the upregulated glycolysis [26,27]. NADPH is an essential cofactor for replenishing reduced glutathione (GSH) which is a critical antioxidant. Therefore, not only are cancer cells’ multiple urgent requirements catered to but cancer cell oxidative stress is also controlled by the Warburg effect [8]. Tumor cells have been reported to switch between the isoforms of pyruvate kinase, used in the last steps of glycolysis [28]. PKM2 the isoform found in high levels in tumor cells is slower and leads to the accumulation of PEP which in turn activates PPP by feedback inhibition of the glycolytic enzyme triosephosphate isomerase (TPI). This produces more NADPH which reduces ROS and further amplifies the inhibitory effect of PKM2 [26,27], Therefore ROS and PKM2 form a negative feedback loop to maintain ROS in a tolerable and functional range. The ROS-regulated gene, hypoxia-inducible factors (HIF-1α) regulates hypoxia-associated genes, some of which are associated with the Warburg effect and its accompanying pathways and hence, are a target of cancer therapies. PKM2 has been found to be the prolyl hydroxylases (PHDs)-induced coactivator for HIF-1α [8,29]. HIF-1α also regulates the MYC proto-oncogene which produces MYC protein [30] that regulates genes participating in energy generation and cell growth and proliferation. HIF-1α and MYC activate hexokinase 2 (HK-II) and pyruvate dehydrogenase kinase 1 (PDK1), which inhibits TCA and increases conversion of glucose to lactate [31]. Glucose transporter 1 (GLUT1) and lactate dehydrogenase A (LDHA) are also activated by HIF1 and MYC independently, resulting in increased glucose influx and higher glycolytic rates [13]. Warburg effect increases steady-state ROS condition in cancer cells by producing lactate that is extruded through monocarboxylate transporters to the microenvironment of cancer cells which has no antioxidant properties in contrast to pyruvate, citrate, malate, and oxaloacetate together with the reducing equivalents (NADH.H^+^) which are antioxidant intermediates. This increased oxidative stress in cancer cells is stopped from reaching cytotoxic levels by some antioxidant effects exerted by hexokinase II (HK II) and NADPH.H^+^ produced through HMP shunt. Latest studies show tumor cells have the capability to carry about both glycolytic and oxidative phosphorylation (OXPHOS) metabolism which makes them resistant to oxidative stress through enhanced antioxidant response and increased detoxification capacity [32]. The changes related to energy metabolism may be correlated to the expression of certain p53 downstream genes regulated by it, including SCO2, TIGAR, and the p53 inducible gene 3 (PIG3) [33,34,35].

### 2.3. NADPH Oxidase, Cox, and Xanthine Oxidase Produce ROS

The NADPH oxidases NOX catalytic subunit carries about the transfer of electrons from NADPH to the molecular oxygen producing ROS as their primary function [36]. The other oxidases like the mitochondrial electron transport chain produce superoxide (O_2_^•–^) as a by-product of another oxidative reaction. Furthermore, xanthine dehydrogenase gets converted to xanthine oxidase which is a dysfunctional variant of the parent enzyme which generates uncoupled eNOS. The kinetics of ROS formation and the nature of the ROS produced are different in the four nonphagocytic NADPH oxidase isoforms. Electrons transfer across the biological membranes through NOX and produce O_2_^−^ which gets rapidly converted to H_2_O_2_ [17]. This H_2_O_2_ after diffusing across the membrane can affect multiple cellular signaling events. Increased NOX-derived ROS in cancer cells affects two major characteristics of cancer progression, i.e., stimulation of cell survival and genomic instability. H_2_O_2_ activates MAPK signaling, neutrophil phagocytosis, apoptosis, cellular senescence, and cell growth. It also plays a significant role in oxygen sensing and under hypoxic conditions, it stimulates the release of hypoxia-inducible factor (HIF-1α) and then vascular endothelial growth factor (VEGF) thus promoting angiogenesis [37].

### 2.4. ER Stress Leads to ROS 

**Figure 1:**
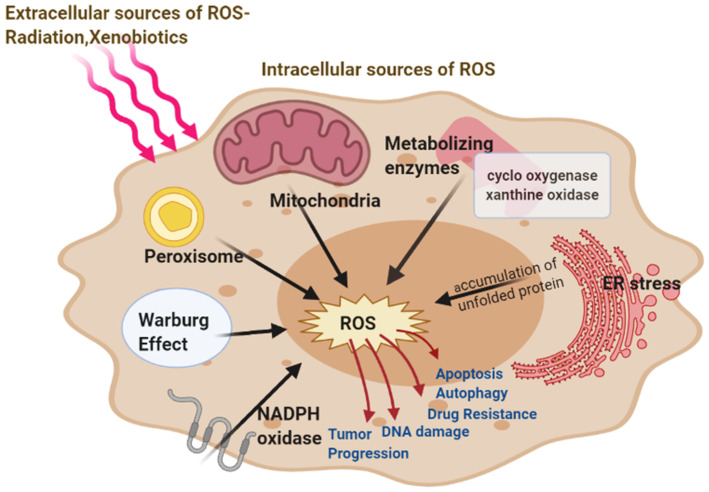
Major intracellular Sources of ROS-mitochondria, peroxisome, endoplasmic reticulum (ER) stress, nicotinamide adenine dinucleotide phosphate hydrogen (NADPH) oxidase, metabolizing enzymes, and extracellular (Radiations, Xenobiotics) sources of reactive oxygen species (ROS) generation. ROS involved in cancer resulting in the development and progression of the disease.

In the endoplasmic reticulum, the catalytic processes of oxidoreductase Ero1 and NADPH oxidase (NOX) produce ROS. The major source of cellular ROS is the oxidative protein folding carried about by Ero1 which uses the oxidative power of molecular oxygen to initiate redox relays which ultimately leads to disulfide bond formation in the newly folded proteins. The luminal H_2_O_2_ arising from Ero1 and NOX are scavenged by ER peroxidases, such as peroxiredoxin 4 (Prx4), as well as the glutathione peroxidases GPx7 and GPx8 and thereby prevent H_2_O_2_ leakage from the ER [38]. The accumulation of unfolded proteins, i.e., persistent ER stress leads to redox-amplified imbalances in the Ero1/PDI electron flow increasing production of ROS at the ER which can be counteracted by an influx of reduced glutathione (GSH) [39]. The production of ROS activates the unfolded protein response (UPR) inactivating the sulfhydration of protein tyrosine phosphatase 1B (PTP1B). This results in increased phosphorylation of PKR-like endoplasmic reticulum kinase (PERK) thereby activating it. PERK plays an important role in restoring cellular homeostasis by regulating a switching mechanism between autophagy and apoptosis [40]. ROS-mediated ER stress also signals activation of Nrf2 antioxidant response, thereby increasing stress resistance and lifespan [41,42]. ROS can leak through the ER through the aquaporin 8, the ER ROS pore. Similarly, Peroxisomal ROS production can leak into the cytosol, and lead to oxidation of important signaling molecules like the NF-kB and PTEN (Figure 1).

## 3. Mechanism of Oxidative Stress-Related Carcinogenesis

ROS provokes programmed cell death in normal cells. ROS levels may also activate redox-sensitive transcription factors that enhance tumor formation like the Forkhead box class O (FoxO) transcription factors which are activated in response to increased ROS levels and translocated into the nucleus through the cJun N-terminal kinase-dependent signaling pathway. FoxO activation leads to the expression of cellular proteins that serve as ROS scavengers and also regulate a wide variety of additional cellular functions, such as proliferation, apoptosis, and differentiation, that may promote tumorigenesis and cancer progression [43]. It is also possible that through FoxO3, increased ROS levels, induced during chronic inflammation, promote aberrant self-renewal in tumor cells. Tumor cells express catalase in surplus and produce huge concentrations of H_2_O_2_. In this way, the tumor clone itself escapes the toxic action of H_2_O_2_ and destroys neighboring healthy cells [44]. We have discussed below, how ROS increases tumorigenesis by a variety of mechanisms: inducing DNA damage, inflammation, evading immune response, regulating signaling pathways controlling autophagy and apoptosis, angiogenesis, and drug resistance.

### 3.1. Role of ROS in Tumor Cell Proliferation, Survival and Tumor Progression

**Figure 2:**
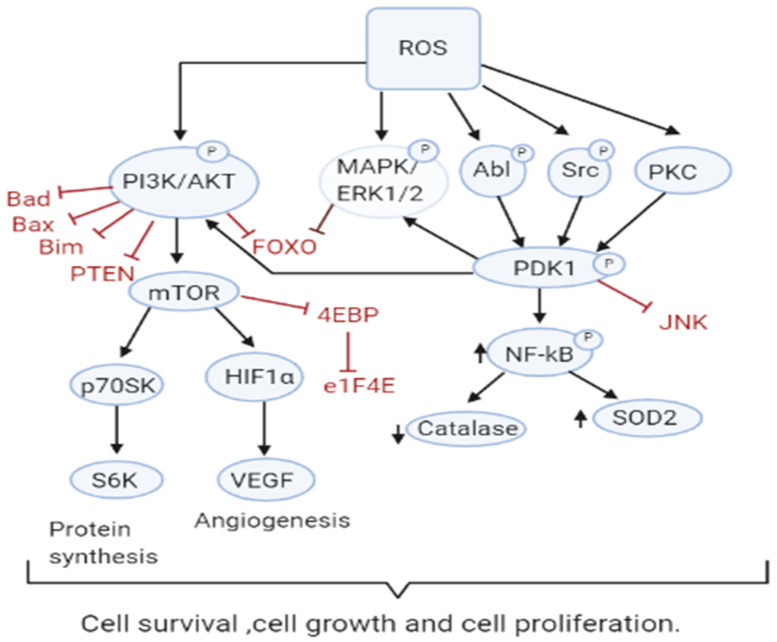
ROS Drive Mitogenic Signaling Cascades. Increased ROS levels contribute to sustained cell survival and proliferation through many pathways including PI3K/AKT, MAPK/ERK1/2, and PKD. ROS also inactivate their downstream targets including Bad, Bax, Bim, Foxo, and PTEN and the JNK pathway.

Increased ROS is responsible for the oxidation of negative feedback loop controllers and hence control the actions of other signaling pathways in tumor growth and programmed cell death by the phosphoinositide 3-kinase/protein kinase B (PI3K/PKB) and mitogen-activated protein kinase (MAPK) signaling pathways [45,46] (Figure 2). Reactive oxygen species generation in cancer cells leads to the inactivation of PTEN that leads to an increase in PI3K/Akt signaling that promotes proliferation. Moreover, the cancer cell cycle progression is promoted when ROS inhibits phosphatase Cdc14B resulting in the activation of cyclin-dependent kinase 1 (Cdk1). As mentioned earlier, the major ROS-regulated gene HIF activates PDK1 that further activates Akt which inhibits the tuberous sclerosis complex (TSC). This downregulates mTOR which is a major regulator of cell growth by controlling mRNA translation, ribosome biogenesis, autophagy, and metabolism [44,47]. MAPK/ERK1/2 are activated by growth factors and K-Ras stimulated pathways, lead to increased cellular proliferation in cancer cells [48]. H_2_O_2_ has also been found to be responsible for the activation of ERK1/2 and pro-survival PI3K/Akt signaling pathway, resulting in increased proliferation [49,50]. Studies on breast, leukemia, melanoma, and ovarian cancer have shown ERK1/2 plays additional roles like cell survival, anchorage-independent growth, and motility [51]. The Akt pathway inactivates pro-apoptotic Bad, Bax, Bim, and Foxo transcription factors by phosphorylation thereby promoting cell survival [52,53]. Akt is activated by the Epithelial growth factor (EGF)-derived H_2_O_2_ production, observed in ovarian cancers [54]. Cell survival is promoted by the oxidation and inactivation of the negative regulators of PI3K/Akt signaling, i.e., the phosphatases PTEN and PTP1B. The tumor suppressor PTEN has been found to be reversibly inactivated by H_2_O_2_ in a variety of cancers [55,56].

PKD signaling plays an important role in the detoxification from elevated ROS production and stimulation of anti-apoptotic genes [57,58,59]. PKD1 signaling leads to upregulation of NFκB which also plays an important role in the proliferation and survival of the cell. PKD1 also promotes cell survival through activation of ERK1/2 and down-regulation of the pro-apoptotic c-Jun N terminal protein kinase (JNK) pathway [60]. An increase in antioxidants SOD2 and Nrf2 has been observed, however, catalase levels appear to decrease providing a role for PKD1 in cell survival [61]. Other members of the PKD family, PKD2 and PKD3 are implicated to play a role in various other cancers [60]. The tumor suppressor genes produce proteins that play important roles as antioxidants. For instance, p53 could regulate the expression of various antioxidant enzymes including catalase, SOD2, and GPX1 thereby decreasing ROS accumulation [62]. However, since p53 is lost or mutated in most cancers, ROS accumulation and pro-tumorigenic signaling is found.

### 3.2. Role of ROS in Apoptosis-Tumor Suppressive Role

Though ROS activates mitogenic signaling pathways, high levels of ROS have the ability to induce cell cycle arrest, senescence, and cancer cell death either by the initiation of intrinsic apoptotic signaling in the mitochondria or by extrinsic apoptotic signaling by the death receptor pathways [63]. ROS induces apoptosis by activating ASK1/JNK and ASK1/p38 signaling pathways in human cancer cells [64,65]. These pathways are activated when TRX1 is oxidized by H_2_O_2_ which subsequently dissociates from ASK1, thereby activating the downstream MAP kinase kinase (MKK)4/MKK7/JNK and MKK3/MKK6/p38 pathways leading to suppression of anti-apoptotic factors [17,66,67,68]. It has also been shown that ROS mediate the downregulation of FLICE inhibitory proteins (FLIP proteins) by ubiquitination and subsequent degradation by the proteasome and thereby induce apoptosis by Fas ligand activation [69]. Collectively, these observations support a tumor-suppressive role of ROS [70]. Recent studies have shown that p53 plays an important role in oxidative stress-related cell death. A regulatory signaling protein of phosphatidyl-3-OH kinase (PI (3) K), p85, participates in the cell death induced by oxidative stress independent of PI (3) K [71]. This protein p85 is upregulated by p53. Sir2α has been found to interact with p53 and attenuate p53-mediated functions and hence is a potential cancer therapeutic target [72].

JNK pathway activation by elevated ROS production results in apoptosis initiated by intrinsic apoptotic signaling through mitochondria or extrinsic apoptotic signaling mediated by death receptor pathways [73,74,75]. JNK pathway mutations have been found to be inactivated in various cancers suggesting that these pathways may be implicated in apoptotic signaling [76]. The activity of apoptotic effectors including the Bcl-2 family of proteins and cytochrome c are affected by the overproduction of ROS leading to the activation of the caspases, a prominent hallmark of apoptosis, resulting in the cleavage of poly ADP ribose polymerase (PARP), DNA fragmentation, and cell death [77].

**Figure 3:**
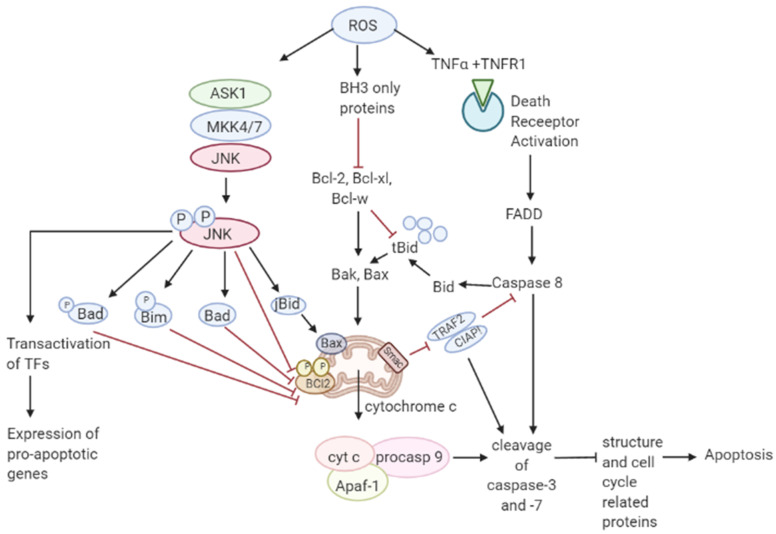
Role of ROS in apoptosis. Toxic ROS levels damage the mitochondrial membrane releasing cytochrome c to the cytoplasm which forms a complex with Apaf-1 and pro-caspase 9. This induces the cleavage of caspase-3 and -7 resulting in apoptosis. Additionally, binding of TNFα ligand to TNFR1 death receptor triggers the activation of caspase 8 leading to cleavage of caspase 3. Caspase 8 activation also cleaves Bcl-1 protein Bid to form tBid which further leads to the release of cytochrome c in the intrinsic apoptotic pathway.

As mentioned in Figure 3, elevated ROS can also result in apoptosis by binding of ligands to death receptors which trigger caspase activation of the initiator caspase 8 leading to cleavage of downstream caspase 3 and Bcl-2 protein Bid to tBid which then translocate into the mitochondria causing the release and translocation of cytochrome c [78,79]. Cytochrome c forms a complex with apoptotic protein-activating factor 1 (Apaf-1) and pro-caspase 9 inducing the cleavage of downstream caspase-3 and -7. Members of the Bcl-2 family, anti-apoptotic (Bcl-2, Bcl-w, and Bcl-xL) are inhibited, and pro-apoptotic (Bad, Bak, Bax, Bid, and Bim) are activated in apoptotic signaling [80]. The loss of cytochrome c from the mitochondria will disrupt and damage the mitochondrial ETC and further cause elevated production of ROS [81]. ROS-induced apoptosis can be attributed mainly to decreased GSH levels and the loss of redox homeostasis [82].

### 3.3. Role of ROS in Autophagy-Both Tumor Suppressive and Tumor Promoting Roles

**Figure 4:**
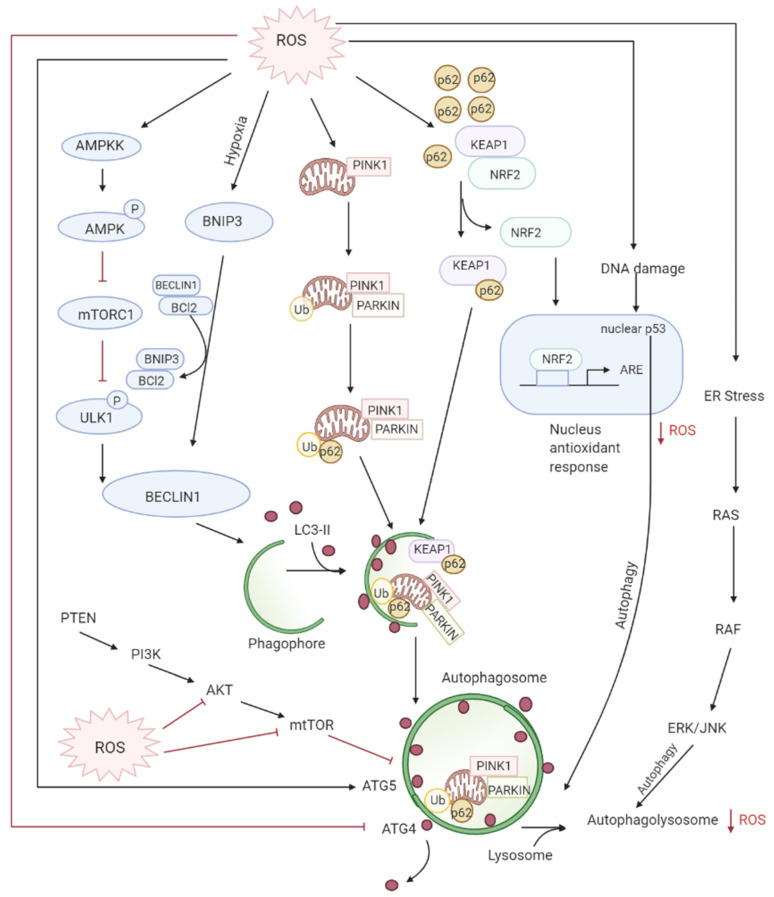
ROS levels regulate autophagy levels by different pathways: firstly, oxidation of ATG4 leads to accumulation of autophagosomes, secondly, the AMPK signaling cascade induces autophagy through the ULK1 complex. Thirdly, the disruption of BCl-2-BECLIN interactions also initiates autophagy. Lastly, the alteration of mitochondria homeostasis leads to mitophagy activation which checks ROS accumulation by elimination of damaged mitochondria. The degradation of KEAP1 by selective autophagy mediated by p62 leads to the expression of Nrf2-regulated antioxidant genes thereby reducing ROS.

Autophagy is the controlled lysosomal pathway that regulates cellular homeostasis by degradation and recycling of proteins and organelles within a cell [83]. ROS regulates autophagy in both direct and indirect ways. Direct regulation involves modification of key proteins like Atg4, Atg5, and Beclin which are involved in the autophagy process. Indirect regulation by ROS involves alteration of signaling pathways that can induce autophagy such as the JNK, p38. ROS have also been found to inhibit Akt signaling and downstream mTOR and thereby induce autophagy [84]. Autophagy is one of the first defenses against oxidative stress damage and is upregulated in response to elevated ROS levels [85]. Autophagy has been found to be regulated by the mammalian target of rapamycin complex1 (mTORC1) and its upstream activators PI3K and AKT that suppress autophagy whereas negative regulator of PI3K and AKT pathways PTEN has been found to induce autophagy [86]. DNA damage caused by the ROS produced by mitochondria leads to activation of p53 that has been documented to regulate autophagy [87] (Figure 4).

Deregulated autophagy has been found to have a role both in tumor progression and tumor suppression. During the early steps of cancer development, autophagy inhibits tumorigenesis by preventing ROS-induced damages on DNA and protein. However, during the later stages of cancer development (promotion, progression, and metastasis), autophagy plays a pro-tumoral role by eliminating ROS-induced metabolic stress and producing nutrients required for cancer cell survival. The cancer cells under hypoxia induce the formation of ROS which can activate autophagy in neighboring stroma cells which then provide high-energic nutrients, such as lactate or ketones, necessary for cancer cell survival and proliferation in accordance with what we have seen earlier, also termed as “tumor-stromal co-evolution”.

Increased ROS production induces autophagy of damaged mitochondria called mitophagy restoring the physiological ROS levels. [88] This selective autophagy is mediated by two different molecular pathways: NIX/BNIP3L and PARKIN (PARK2)/PTEN induced putative kinase 1 (PINK1) [89,90,91,92]. Nix/BNIP3L targets mitochondria for degradation after interacting with GABARAP and GABARAPL1 at the autophagosome [93,94]. Whereas selective degradation of damaged dysfunctional mitochondria occurs through PARKIN/PINK1 after ROS induces mitochondrial membrane depolarization [92]. Moreover, Nrf2/Keap1 and SQSTMI/p62 pathways also regulate mitophagy by decreasing ROS [95]. SQSTM1/p62 interacts with Nrf2/Keap1, forming a complex with Keap1, preventing Nrf2 degradation resulting in the release and translocation of Nrf2 to the nucleus where it activates antioxidants [96,97].

Studies have proven that elevated ROS levels can also lead to defective autophagy. Deletion of autophagy genes ATG5 or ATG7 leads to autophagy inhibition and accumulation of damaged mitochondria which results in chronic oxidative stress, tissue damage, and inflammation which all favor tumor initiation [97,98,99]. BECLIN1 (ATG6/BECN1) which is an essential gene in autophagy as well as a tumor suppressor has been found deleted in various cancers, resulting in damage to mitochondria, oxidative stress, and disease progression [100,101,102]. Furthermore, during later stages of tumor initiation, autophagy is required for cell transformation by the RAS oncogene in order to promote cell tolerance to stress, therefore for Ras-induced tumorigenesis, active autophagy is necessary to maintain cellular homeostasis [83].

### 3.4. ROS and Inflammation

The dynamic role of chronic inflammation in cancer has long been established. The presence of inflammatory cells in the cancer cell’s environment enhances their proliferation potential as it is abundant in ROS and RNS promoting DNA damage, upregulation of growth factors and cytokines, and growth-supporting genes along with inactivation of apoptosis [103]. Inflammatory cells further produce more ROS/RNS by inducing oxidant-generating enzymes such as NADPH oxidase, iNOS, xanthine oxidase (XO), and myeloperoxidase (MPO) which further add to the mutation load by damage to DNA, RNA, Lipids, and nitration and oxidation of proteins [1,104]. Inflammatory tissues also release cytokines that activate NFκB, which stimulates COX2, lipoxygenase (LOX), and iNOS, resulting in overproduction of ROS and RNS [104]. These in turn stimulate oncogenes such as c-Jun and c-Fos, the overexpression of which is associated with many cancers. Inflammation promotes cancer initiation and progression via vascularization and remodeling of TME, which is an essential step in tumor cell survival.

As seen earlier, an increase in mitochondrial ROS leads to apoptosis by the TNF-initiated death signal after activating JNK and induction of mitochondrial outer-membrane permeabilization [105,106]. It has been found that O_2_^•−^ generates H_2_O_2_ after reacting with manganese SOD (MnSOD) in the mitochondrial matrix, which activates redox-sensitive transcription factors such as HIF-1α and NFκB and pro-inflammatory cytokines, as well as inflammasomes [107]. These complexes work by activating inflammatory caspases (caspase-1 and -12) and cytokines (IL-1β and IL-18) in macrophages [108]. An increase in ROS inside the cell by NADPH oxidase or mitochondrial ETC causes the redox-sensitive protein thioredoxin (Trx)-binding protein-2 (TBP-2) or TXINP (thioredoxin Interacting protein) to dissociate from Trx enabling the binding of TXINP and NLRP3. NLRP3, a redox-sensitive inflammasome gets activated on interacting with another due to increased intracellular ROS generation [109]. This NLRP3 Inflammasome then leads to activation of caspase-1 and IL-1β and IL-18. MtROS activate TNF-α-converting enzymes which cleave receptor–1 (TNFR1) which is important for inflammatory progression [110]. NADPH oxidase NOX4 also enables ROS generation in response to Inflammatory stimuli LPS, TNF α, hyperoxia, TGF-β, and hypoxia [111]. ASK1 and ASK2 have been found to play a role in ROS-mediated carcinogenesis via MAPK signaling cascades by induction of inflammation and apoptosis [112].

Oxidative stress and inflammatory response always reinforce each other in the tumor progression. One of the important promoters of tumor development is chronic inflammation which is majorly caused by Nuclear factor-κB (NFκB) activation [113]. The transcription factor, NFκB, plays important role in proliferation, cell survival, regulation of the cell cycle, and the development of resistance to drug therapies [114,115]. It is a sensitive sensor of oxidative stress and detects H_2_O_2_ at a low level. ROS-mediated activation of mitogen-activated protein kinases (MAPKs) contributes to the production of inflammatory mediators including pro-inflammatory factors like Tumor-necrosis factor (TNF-α) and interleukin-6, interleukin (IL)-1β, and transforming growth factor (TGF)-β which act as mediators leading to activation of NFκB and thereby suppression of cell death and stimulation of cell proliferation. NF-𝜅B expresses proinflammatory cytokines and chemokines and is responsible for the expression and activity of cyclooxygenase 2 (COX2) [116]. Oxidative stress, the NFκB pathway, and the JAK-STAT pathway work together in cancer progression. Oxidative stress stimulating the NFκB pathway generates more ROS which in turn increases oxidative stress. However, inflammatory mediators block the inflammatory process by stimulating the suppressors of cytokine signaling (SOCS), linked to JAKs, interrupting the JAK-STAT pathway [26,117]. NF-kB, a collection of the Rel family of transcription factors, inhibits apoptosis by upregulating several antiapoptotic genes. Nrf2, a transcription factor, is activated in tumor cells to increase the production of antioxidant proteins to maintain the redox balances in the body. Kelch-like ECH-associated protein 1 (Keap1) negatively regulates Nrf2. It has been shown in studies that the activation of Nrf2 can reduce oxidative stress and inflammatory response as Nrf2 dissociates from Keap-1 after its degradation through the ubiquitin-proteasome pathways and travels to the nucleus to activate the antioxidant response elements (AREs) leading to increased activity of antioxidants including catalase, GPXs, PRXs, and glutathione synthesis [118]. Deregulation of the Nrf2 pathway and mutations in Keap1 has been associated with various cancer.

As seen earlier, the hypoxic microenvironment of cancer cells induces autophagy via increased ROS production and subsequent JNK activation. NF-kB downregulates JNK activation by suppression of TNF-α-induced ROS accumulation [119]. The apoptosis in cancer cells is prevented by NFκB activation by upregulating the expression of anti-apoptotic genes, such as those encoding Bcl-XL (B-cell lymphoma XL), BFL1 (a Bcl-2-related protein), and GADD45β (growth arrest and DNA-damage-inducible 45β). ROS have therefore been shown to act as immunosuppressive agents in cancer microenvironments facilitating tumor invasion and metastasis acting not only as oxidative stress mediators but also immune regulators in cancer development.

### 3.5. ROS and DNA Damage

Significant studies have shown that ROS interacts with cellular macromolecules such as DNA, proteins, and lipids interfering with vital cellular functions. ROS causes oxidative modifications such as DNA base alterations, strand breaks, damage to tumor suppressor genes, and expression of proto-oncogenes resulting in the transformation of normal cells into malignant cells. One of the most abundant oxidative DNA lesions produced is 8-hydroxydeoxy guanosine (8-OHdG), which is mutagenic [120] and is found elevated in various human cancers. The transcription factor Nrf2 controls the expression of antioxidant enzyme genes and also genes that control immune and inflammatory responses, carcinogenesis, and metastasis. It combats oxidative stress by induction of cytoprotective enzymes, such as GST, GPx, and oxidoreductases. Cancer patients exhibit disrupted Nrf2-Keap1 interaction through somatic mutations [121,122]. BRCA1 a tumor suppressor gene found mutated in many cancers, is a caretaker gene, responsible for repairing DNA ultimately helping the cells to cope with oxidative stress [123,124]. It controls the activity of the transcription factors Nrf2 and NFκB and hence can upregulate several genes involved in the antioxidant response. The redox factor 1/AP endonuclease 1 (Ref1/APE1) has also been found to reduce the generation of ROS in breast cancer cells [125]. Ras activation in tumors has been associated with point mutations and has been observed in 30% of the tumors [126]. The Ras gene family includes G proteins, Ha-ras, N-ras, and Ki-ras, which participate in cell signaling and mutations in this oncogene render the proteins constitutively active [127,128]. Mutant Ras has been found to increase mitochondrial mass and ROS levels leading to DNA damage contributing to transformation [129]. Mutant Ras produces H_2_O_2_ by upregulating the Nox4-p22phox, making Nox4 a critical mediator of oncogenic Ras-induced DNA damage [130]. DNA strand breakage and levels of peroxides have been found to increase significantly with the activation of mutant K-ras in non-transformed epithelial cells. Of the three mitochondrial sirtuins, Sirt3 which belongs to a class of proteins that possess histone deacetylase has been linked to longevity in humans, acting as a tumor suppressor protein [131]. The expression of an oncogene, Myc or Ras, in Sirt3 enhances ROS production by increasing glycolysis and decreasing oxidative phosphorylation. Under hypoxic conditions loss of Sirt3 increases tumorigenesis in cancer cells in a ROS-dependent manner by the activation of by HIF-1α [132]. Oxidative stress and antioxidative stress genes that are considerably altered in tumor cells include-GPX8, ATOX1, PRDX2, PRDX6, PTGS1, SEPP1, and DEFB122 that are upregulated, while there was a decrease in expression of SIRT2, TTN, CYBA, UCP2, and AKR1B1 [133]. TNF-α may also play an important role in tumor initiation by stimulating the production of intracellular ROS that may damage DNA and lead to genomic mutations [134]. A study exogenously applied ROS-induced G1 arrest in proliferating fibroblasts showing that oxidative stress could play a role in the accumulation of p53 and the activation of cdc2 [135]. Increased ROS levels are associated with the inactivation of certain genes like FoxO3, TP53, and ATM [136]. The tumor suppressor p53 gene TP53 has been found to be significantly and progressively downregulated in cancer cells caused by the excessive oxidation of DNA. The TP53 gene plays an important role in protecting the genome from oxidation by ROS similarly the ataxia telangiectasia mutated (ATM) gene mediates the cellular response to DNA and oxidative damage. The FoxO3 gene decreases ROS levels by influencing the regulation of ATM [24]. Research is being carried out to relate polymorphisms in antioxidant genes to cancer progression as it can lead to altered enzyme activity. Damage to DNA repair enzymes is also associated with an increase in the level of oxidative DNA damage.

### 3.6. ROS-Mediated Alterations in Protein Stability and Lipid Peroxidation

ROS-mediated cell signaling has been implicated to cause certain protein modifications for instance the sulfhydryl (-SH) group of cysteine residues in proteins are modified to their oxidized derivatives, as well as causing changes to occur in the conformation by the formation of intramolecular disulfide bridges which alters the protein activity. However, the impact of protein redox modifications depends on the proteins’ biochemical properties and three-dimensional arrangement, as well as on the abundance and kind of reactive oxygen species (ROS) [137].

The reduced state of critical cysteines in some transcription factors appears to promote DNA binding and transactivation mediated by disulfide-reducing systems (such as thioredoxin) and redox factor-1 (Ref-1) [138]. Intermolecular disulfide linkages can mediate protein dimerization for instance protein kinase (PK) dimerization may lead to its dissociation from an inactive complex. Protein crosslinking can be induced by H_2_O_2_ or peroxidase-catalyzed di tyrosine formation [1,134]. A potential mechanism for ROS-mediated alterations in protein stability is by targeting the transitional metal-containing proteins by mixed-function oxidases which target them for ubiquitination and degradation by proteases. ROS attack proteins producing carbonyls and other modifications in several amino acids. These changes on receptors, signal transduction proteins, enzymes, and transporters impair the function of these proteins [139]. Some damage to proteins can be repaired while proteins with irreversible damage are destroyed and replaced with new ones. Lipids are also oxidized by ROS, and this is known as ‘lipid peroxidation’, which results in a reduction in membrane fluidity, an increase in membrane permeability, and damage to membrane proteins. At low lipid peroxidation, the cells stimulate antioxidants defense systems promoting tumor survival, whereas, under medium or high lipid peroxidation conditions, the cells induce apoptosis or necrosis programmed cell death; both of which cause molecular cell damage facilitating the development of various pathological states [140].

Therefore, lipid peroxidation plays a role in carcinogenesis but its end products such as malondialdehyde, 4-hydroxy-2-nonenal, and isoprostanes, could be used as biomarkers and act as a potential therapeutic strategy in case of colorectal cancer [141]. Oxidized lipids also lead to changes in the physical characteristics of biomembranes at large including bilayer thickness, polarity, and thermal phase behavior. These changes result in augmented permeability, loss of lipid symmetry, and fast lipid trans bilayer diffusion (flip-flop); which further interfere with lipid-protein interactions, leading to changes in metabolic pathways, inflammation, and apoptosis [142].

### 3.7. Adaptation of Cancer Cells to ROS

Mesenchymal stromal cell (MSC) metabolism has the ability to modify cancer cell metabolism and alter malignancy by transferring mitochondria and/or mitochondrial DNA (mt DNA) to cancer cells thereby increasing mitochondrial content and enhance oxidative phosphorylation (OXPHOS) to favor proliferation and invasion [143]. The stromal metabolism by the cancer-associated fibroblasts produces high-energy nutrients (such as lactate and ketones) that act as fuels for mitochondrial biogenesis [6]. In this way cancer cells maintain a pool of functional mitochondria by coupling mitophagy to mitochondrial biogenesis and also from nonmalignant cells in the tumor microenvironment by forming intercellular tunneling nanotubes (TNTs). Cancer cells follow “tumor-stroma co-evolution” by secreting hydrogen peroxide in adjacent fibroblasts and other stromal cells, which mimics the effects of hypoxia, under aerobic conditions, resulting in excess production of reactive oxygen species (ROS) [6,144]. Therefore, oxidative stress initiated in tumor cells is transferred to cancer-associated fibroblasts laterally and vectorially via Hydrogen peroxide. Excess stromal production of ROS drives the onset of antioxidant defense in adjacent cancer cells, protecting them from apoptosis.

Cancer cells also have mechanisms of immune evasion of ROS through the antioxidant defense. During hypoxia when there is a high production of ROS and NO•, glutathione maintains intracellular redox homeostasis. Glutathione reductase enzyme (GRd) transforms and recycles ROS, by converting the oxidized state of glutathione GSSG to the reduced state GSH, taking electrons from NADPH mainly derived from the pentose phosphate pathway. A decrease in the ratio of GSH to GSSG values is indicative of oxidative stress [145].

Another master regulator of the antioxidant pathway is the transcription factor, Nuclear factor erythroid 2-related factor 2 (Nrf2) which in normal conditions remains bound to its inhibitor Kelch-like ECH-associated protein 1 (Keap1). ROS reacts with redox reactive cysteines in Keap1, releasing Nrf2 into the nucleus where it binds to ARE leading to the expression of genes involved in the cellular antioxidant defense. Inhibition of pro-inflammatory responses of Cox-2 and iNOS expression has been observed with the activation of Nrf2. Nrf2 is inhibited by modifications of cysteine residues of Keap1and provides cytoprotective effects against Fas-mediated apoptotic pathways [146]. Karyopherin-6 (KPNA6) facilitates nuclear import and attenuates Nrf2 signaling and restores Nrf2 protein to basal levels [147]. Nrf2 impairment leads to oxidative stress, inflammation, and mitochondrial dysfunction [148]. Nrf2 is a tumor suppressor however hyperactivation of Nrf2 creates an environment that favors the survival of both normal and malignant cells, protecting them against oxidative stress [149]. Carbonyl reductase 1 is another important enzyme that regulates the expression of Nrf2 during oxidative stress and helps to detoxify ROS [26].

### 3.8. Role of ROS in Drug Resistance

The increased ROS production through induction of pro-tumorigenic signaling, enhanced cell proliferation and survival, increased genetic instability and DNA damage, and metabolic adaptations, contribute to drug resistance and hence further progression of cancer. Accumulation of multiple mutations caused by the overproduction of ROS results in an increased risk of tumor cells developing resistance to therapies used. Drug resistance is observed in both AML and CML which is associated with mutations in receptor tyrosine kinases, namely FLT3-ITD and Bcr-Abl. Resistance to the frequently used protein tyrosine kinase inhibitors, midostaurin, and imatinib can be attributed to the DNA oxidation caused by NOX4-generated H_2_O_2_ downstream of the pro-survival PI3K/Akt pathway [150,151]. When tumor cells adapt to mitochondrial malfunction it has been found to be linked to drug resistance. To resist chemotherapy, tumor cells adapt to hypoxia and respiratory injury through the activation of glucose metabolism and therefore become less sensitive to it [152]. Radiation therapy resistance can be attributed to the Hypoxia-induced accumulation of HIF-1α [153,154,155].

### 3.9. Role of ROS in Angiogenesis

High levels of reactive oxygen species (ROS) such as superoxide and H_2_O_2_ have been found to function as signaling molecules to mediate various growth-related responses including angiogenesis and mutagenesis. Endogenous antioxidant enzymes such as SOD and thioredoxin regulate ROS-dependent angiogenesis. An important angiogenesis growth factor is VEGF that stimulates permeability, proliferation, migration, and tube formation of ECs primarily through the VEGF receptor type2 (VEGR2, KDR/Flk1) [156,157]. Oxidative stress can increase VEGF expression in tumor cells, which is seen to increase microvessel counts and poor prognosis in cancers [158]. Hypoxia in the tumor microenvironment stimulates the induction of VEGF which stimulates NADPH oxidase. Nox produces ROS which further induces oxidative inactivation of protein tyrosine phosphatases (PTPs) and PTEN to promote VEGFR2 auto phosphorylation activating redox signaling events like c-src, Akt, eNOS, p38MAPK, ERK1/2 [158]. A considerable amount of ROS is generated by NADPH oxidase which includes Nox1, Nox2, Nox4, Nox5, p22phox, p47phox, and the small G-protein Rac1. This oxidase produces ROS which is involved in diverse redox signaling pathways inducing transcription factors and angiogenesis genes. Nox isozymes have been shown to increase in association with ROS production out of which Nox1 is highly overexpressed in human colon cancers and prostate cancers [159]. Nox1-induced H_2_O_2_ increases VEGF and VEGFR expression and MMP activity, markers of the angiogenic switch, thereby promoting vascularization and rapid expansion of the tumors [160]. Studies have shown increased expression of Nox4 and Nox5 in melanoma cells and prostate cancer cells respectively [161,162]. Nox1 redox signaling has been found to be controlled by mitochondria and the loss of control of this signaling contributes to tumorigenesis. The redox-sensitive transcription factors HIF-1α, p53, Ref1, NFĸB, and Ets activate the expression of redox-sensitive genes such as VEGF, MMP, uPA, PAI-1. Trx-1 is a validated cancer drug target that is involved in many of the hallmarks of cancer including increased proliferation, resistance to cell death, and increased angiogenesis [163]. Recent studies have shown that overexpression of a gene SIRT6, through regulating HIF-1α, promoted invasion, migration, proliferation, and angiogenesis [164].

### 3.10. Role of ROS in Metastasis

The effects of ROS are not specific to cancer cells and may result in the destruction of normal cells and tissues as well. These changes in the surroundings of cancer cells bring about invasion and adhesion processes. ROS also serve as second messengers in gene regulatory and signal transduction pathways leading to upregulation of the expression of various genes, including those closely involved in the metastasis and proliferation of cancer cells. Various stages of tumor metastasis need upregulation of matrix metalloproteinases (MMPs), adhesion molecules, EGF, EGF receptor (EGFR), and vascular endothelial growth factor. ROS have been found to increase the expression and/or activate these proteins, leading to aggravation of tumor metastasis [165]. EGFR, which plays an important role in tumor metastasis is found to be highly expressed in a variety of tumors, such as breast, colon, gastric, pancreatic, ovarian, and prostate cancers, gliomas, and melanomas [166].

The cell’s defense against ROS includes antioxidant enzymes that detoxify ROS and prevent them from accumulating at high concentrations [167]. Cell detachment during metastasis upregulates PDK4 which inhibits PDH and decreases the flux of glucose carbon into the TCA cycle [168]. Cell detachment also upregulates NFkB which increases the expression of MnSOD, the principal mitochondrial antioxidant enzyme, to detoxify mitochondrial ROS resulting from detachment [169]. Moreover, it has been found that cells depleted of MnSOD are hypersensitive to matrix detachment.

Cancer cells follow “tumor-stroma co-evolution” by secreting hydrogen peroxide in adjacent fibroblasts and other stromal cells, which mimics the effects of hypoxia, under aerobic conditions, resulting in excess production of reactive oxygen species (ROS) [6,136]. Therefore, oxidative stress initiated in tumor cells is transferred to cancer-associated fibroblasts laterally and vectorially via Hydrogen peroxide. Excess stromal production of ROS drives the onset of antioxidant defense in adjacent cancer cells, protecting them from apoptosis. When cancer cells metastasize, they detach from the extracellular matrix which activates pro-apoptotic proteins (e.g., BMF, BIM, and BID) and pro-apoptotic members of the Bcl-2 family of proteins (BAK and BAX), eventually resulting in anoikis, a type of apoptotic cell [170]. Cancer cells increase their metastatic potential by elevating their threshold for anoikis. Cancer cells also purposefully restrain pyruvate from entry into mitochondrial oxidative metabolism as the ROS produced as byproducts of mitochondrial respiration exhibit anti-metastasis activity [171]. Thus, cancer cells gain increased anoikis resistance and survival advantage for metastasis.

## 4. Targeting ROS

ROS has both pro-tumorigenic and anti-tumorigenic signaling which can be manipulated in the treatment of cancer to prevent ROS production or to induce tumor cell death (Table 1).

### 4.1. Targeting Tumor Death by Upregulation of ROS

By increasing the production of ROS levels to toxic levels and exhaustion of the antioxidant system capacity causing programmed cell death, the anti-tumorigenic signaling of ROS can be targeted as a therapy in cancer. Chemotherapy drugs such as anthracyclines, cisplatin, bleomycin, arsenic trioxide increase ROS production resulting in irreparable damage and cell death, and have been used in the treatment of AML, acute lymphoblastic leukemia (ALL), and acute promyelocytic leukemia (APL) [172]. Daunorubicin is an anthracycline that leads to increased activation of sphingomyelinase and ceramide resulting in activation of the JNK pathway leading to apoptosis [173,174]. This is achieved when it reacts with cytochrome p450 reductase [175,176,177] to form semiquinone radical intermediates in the presence of reduced NADPH which further reduces O_2_ to form O_2_^−^ [178,179,180,181,182]. Another widely used anthracycline in the treatment in a broad spectrum of cancers like breast, esophageal carcinomas, endometrial carcinomas, bile duct, pancreatic, gastric, liver, Hodgins and non-Hodgins lymphoma, osteosarcoma, Kaposi’s sarcoma, and soft tissue sarcomas is Doxorubicin [183,184] which works by increasing production of ROS resulting in the activation of the tumor suppressor p53 and ultimately tumor cell death [185,186,187]. Another drug called Sulindac, an NSAID, which works by elevating ROS production has been used to treat colon and lung cancer. It damages the mitochondrial membrane and hence the tumor cells become more sensitive to H_2_O_2_-induced cell death [188].

As mentioned previously, tumor cells adapt to oxidative stress through increased glucose metabolism thereby inhibiting apoptosis through the redox inactivation of cytochrome c [189]. Another approach used in pancreatic and prostate cancer is to use 2 deoxy glucose (2DG) that inhibits glucose causing elevated ROS production leading to cell death [190,191,192]. Studies have shown that 7-formyl-10-methylisoellipticine, an isoellipticine derivative, increases mitochondrial ROS production and induces apoptosis in AML cells with no cytotoxic effects to any organs [193]. A number of chemotherapeutic drugs are currently in use that induces autophagy regulated by ROS.

**Table 1:** List of chemotherapeutic drugs that induce autophagy and/or apoptosis by regulation of ROS [85].

Drug	Target Cancer Type	Primary Action	Secondary Action	Reference
Arsenic trioxide	Ovarian cancer	Induces beclin-1-independent autophagic pathway, modulating SnoN/SkiL expression	Alters TGFβ signaling via ROS generation	[194]
Artemisinin	Cancer cells	Weakens the levels of glutathione, Supply extra ferrous ion to elevate ROS levels	Self-amplification of oxidative stress	[195]
Buthionine-sulfoximine	Cancer cells	Deplete intracellular GSH, may affect STAT3 pathway	Induce oxidative stress	[195,196]
Chloroquine	MCF-7, HT29, U373 cancer cells	Sensitizes cells to hypoxia, due to increased ROS, incapacity to reduce mitochondrial content	Inhibition of autophagy, increases cell death	[197]
Cisplatin	Head and neck cancer	Enhances ROS levels	Induce DNA damage	[198,199]
Curcumin	Colon cancer cells	Induces ROS production, activation of ERK1/2 and p38 MAPK	Autophagic cell death	[200]
Daunorubicin	Breast cancer	Induce ROS, activates the JNK pathway	Lead to apoptosis	[114,201]
Doxorubicin	Breast, esophageal carcinomas, endometrial carcinomas, bile duct, pancreatic, gastric, liver cancer	NO synthase inhibition, Generates ROS, activates p53	Induces tumor cell death	[126,127,128,202]
Diphenylene iodonium	pancreatic cancer	Jak/STAT pathway inhibited, dephosphorylation of AKT/ASK1 pathway	Decrease ROS, lead to apoptosis	[203,204]
Fullerene C60 (Nano-C60)	Normal and drug-resistant cancer cells	Activation of Atg5	Causes autophagy in a ROS-dependent fashion	[205]
Gemcitabine	Head and neck cancer, pancreatic cancer	Activate antioxidant agents, suppress Nox4, block ROS-related signaling pathways, inactivate stromal cells	scavenge ROS	[198,206]
Idarubicin (IDR)	Breast cancer	Induce oxidative DNA damage		[207]
Itraconazole	Liver cancer	Increase ROS	Upregulate expression of death receptor protein FAS, pro-apoptotic protein Bax, decreased expression of anti-apoptotic protein Bcl-2, activating apoptosis	[208]
Medroxyprogesterone	Head and neck cancer	Induction of 15d-PGJ_2_-ligand of PPARγ, increased ROS	Induce apoptosis	[198,209]
Metformin	Pancreatic cancer	Increase MnSOD/SOD2 expression, decrease NOX2 and NOX4 protein expression	Pro-apoptotic effects	[210]
OSU-03012	Hepatocellular carcinoma	Inhibit PDK/AKT signaling pathway inducing apoptotic cell death	ROS accumulation and subsequent autophagic cell death	[211]
Panitumumab (EGFR antibody)	EGFR-expressing metastatic colorectal carcinoma	Increase in GSH levels, reduced stability of proteins	Redox imbalance induced autophagy	[212,213,214]
Proton pump inhibitor, Esomeprazole	Melanoma	Mitochondrial dysfunctions, involvement of NADPH oxidase	Accumulation of reactive oxygen species (ROS)	[215]
Proscillaridin A (PSD-A)	Breast cancer colorectal cancer	ROS generation, Ca^2+^ oscillation	inhibits STAT3 activation, induces apoptosis and autophagy	[216]
Recombinant human HMGB1	Glioblastoma cells	Bind to TLR2 and TLR4, induce NADPH oxidase to produce ROS	activate MAPK and NFκB, release Cytokines	[217]
Resveratrol	Colon cancer cells	Induce ROS and subsequent cytotoxic autophagy	Caspase-8/Caspase-3-dependent apoptosis	[218]
Ruthenium (II) complexes	Cancer cells	DNA damage, Induce ROS	subsequent protective autophagy along with apoptosis	[219]
Suberoylanilide hydroxamic acid (Zolinza, Vorinostat)	Cutaneous T-cell lymphoma, leukemia	Regulate gene expression, Induce ROS	autophagy, prosurvival	[220,221]
Sulforaphane	Therapy-resistant pancreatic carcinoma cell	Promote mitochondria-derived ROS	initiate protective autophagy	[222,223]
Sulindac	colon and lung cancer	mitochondrial damage	elevate ROS production	[129]
Tamoxifen	MCF-7 breast cancer cells	Induced ROS, increased expression of Beclin-1	protective autophagy	[224]
Temozolomide	Malignant gliomas	Suppress ROS/ERK-mediated autophagy	Induce apoptosis	[225]
Valproic acid	Glioma cells	Mitochondrial ROS activates the ERK1/2 pathway	Autophagic cell death	[226]
Vitamin A	Testis tumor Leydig cell lines	Modulate antioxidant enzyme activities	Induce protective autophagy or apoptosis at different doses	[227]
2 deoxy glucose (2DG)	pancreatic and prostate cancer	Disrupt hydroperoxide metabolism, increased glutathione disulfide accumulation, NADP (+)/NADPH ratios	Elevated ROS production leading to cell death	[132,133]
7-formyl-10-methyisoellipticine	AML	Increase mitochondrial ROS production	Induces apoptosis	[134,228]

### 4.2. Targeting Tumor Proliferation by Downregulation of ROS

ROS production in tumor cells can be inhibited in order to suppress pro-tumorigenic signaling, as reduced ROS levels would mean fewer metabolic adaptations and lower levels of DNA damage and genetic instability and therefore decreased cell survival and proliferation. Metformin given to type 2 diabetes patients, is an inhibitor of complex I of the mitochondrial ETC and has also been found to reduce cancer incidence and mortality [229,230]. Metformin shows pro-apoptotic effects by increasing the protein expression of MnSOD/SOD2 and decreased NOX2 and NOX4 protein expression [231]. NOX4 generated ROS production is a potential target in decreasing pro-tumorigenic effects in various cancers which can be suppressed by flavoprotein inhibitor diphenylene iodonium (DPI) resulting in apoptosis via the AKT/apoptosis signal-regulating kinase 1 (ASK1) pathway [232]. Moreover, studies have shown inhibition of the protein tyrosine kinases FLT3-ITD as well as inhibition of p22phox and NOX4 activity in AML cells results in decreased cell survival along with a decrease in DNA damage and genomic instability [233]. Antioxidants have also been thought to reduce ROS production however it is controversial. Few studies on breast cancer have shown that the overexpression of antioxidant SOD3 reduced breast cancer metastasis implicating the use of antioxidants to reduce ROS in cancer therapy [234]. On the other hand, it has also been found that Vitamin A and E and also beta carotene increased the risk of cancer [235,236].

## 5. Conclusions

It has become increasingly apparent that ROS plays an inevitable role in cancer biology. Increased ROS production has become a well-recognized hallmark of various cancers, and an understanding of the pro-tumorigenic and anti-tumorigenic actions of ROS can help analyze at what levels they can be used in tumor-suppressive roles. We have not only been able to positively correlate reactive oxygen species (ROS), to carcinogenesis and to malignant progression of tumor cells; but have also found ROS to promote cell motility and shape the tumor microenvironment by inducing inflammation/repair and angiogenesis. This is because ROS can transduce, as signaling intermediates, contributing to genomic damage and genetic instability. ROS are essential to numerous cellular processes including apoptosis and cell growth as well as regulation of autophagy. A better understanding of how ROS regulates autophagy, as well as apoptosis, opens up opportunities to develop cancer treatment strategies by either induction or inhibition of ROS depending on individual cancer’s molecular context and its microenvironment. It is now possible to develop selective and effective therapies to target cancer cells by studying the role of elevated ROS production in cancer, ROS-regulated signaling pathways, and identifying specific antioxidants as targets. With more research on the subject, we can hope to devise ways where ROS can function as a weapon to target cancer cells specifically without damaging normal cells. Newer methods could be used to evaluate ROS spatial specificities in order to better infer their regulatory mechanisms and downstream influences on different subcellular organelles. Work can be done to elucidate each oxidative modification on tumor growth, survival, and migration in or der to identify cancer-specific redox vulnerabilities which can be exploited to develop cancer therapies. Overall, a better understanding of cancer-specific redox signaling events holds promise in terms of developing tumor-specific cancer therapies without destroying normal cells.

## Abbreviations

2DG	2-deoxyglucose
8-OHdG	8-hydroxydeoxy guanosine
ALL	acute lymphoblastic leukemia
APL	acute promyelocytic leukemia
Apaf-1	apoptotic protein-activating factor 1
Ask1	apoptosis signal-regulating kinase-1
AREs	antioxidant response elements (AREs)
ATM	ataxia telangiectasia mutated
Cdk1	cyclin-dependent kinase 1
COX2	cyclooxygenase 2
CSC	cancer stem cells
DR	death receptor
EGF	epidermal growth factor
EGF-R	epidermal growth factor-receptor
Erk1/2	extracellular-regulated kinases 1/2
FAD	flavin adenine dinucleotide
FLIP	FLICE inhibitory protein
FOXO	forkhead homeobox type O
GF	growth factor
GF-R	growth factor receptor
GSH	glutathione
GSSG	glutathione disulphide
GPX	glutathione peroxidase
GST	Glutathione S-transferase
GLUT1	Glucose transporter 1
H_2_O_2_	hydrogen peroxide
HIF-1	hypoxia-inducible factor-1
HK2	hexokinase 2
JNK	c-Jun N-terminal Kinase
KPNA6	Karyopherin-6
Keap1	Kelch-like ECH-associated protein 1
LDHA	lactate dehydrogenase A
MAPK	mitogen-activated protein kinase
MMP	matrix metalloproteinase
MOMP	mitochondrial outer-membrane permeabilization
mTORC1	mammalian target of rapamycin complex1
mROS	mitochondrial ROS
Nox	NADPH oxidases
Nrf2	Nuclear factor erythroid 2–related factor 2
NFκB	nuclear factor κ-B
NOX	NADPH oxidase
OXPHOS	Oxidative Phosphorylation
PARP	poly ADP ribose polymerase
PDK1	phosphoinositide-dependent kinase 1
PDGF	platelet-derived growth factor
PDGF-R	platelet-derived growth factor-receptor
PEP	Phospho enol pyruvate
PERK	PKR-like endoplasmic reticulum kinase
PKM2	Pyruvate kinase isozyme M2
PTPs	protein tyrosine phosphatases
PTP1B	protein tyrosine phosphatase 1B
PINK1	putative kinase 1
PI3-K	phosphatidylinositol 3-kinase
PKC	protein kinase C
PKD	protein kinase D
PPP	Pentose phosphate pathways
Prdx	peroxiredoxin
PTEN	phosphatase and tensin homolog
ROS	reactive oxygen species
SOD	superoxide dismutase
SOCS	suppressors of cytokine signaling
TGFβ	transforming growth factor β
TIMP	tissue inhibitor of metalloproteinases
TNFα	tumor necrosis factor α
TP1	triosephosphate isomerase
TNTs	intercellular tunneling nanotubes
TrxR	Thioredoxin reductase
VEGF	vascular epithelial growth factor
